# Supplementary material for: Widely assumed phenotypic associations in Cannabis sativa lack a shared genetic basis
Source: PeerJ. 2021 Apr 20;9:e10672. doi: 10.7717/peerj.10672 (PMC8063869; doi:10.7717/peerj.10672)
Supplement: Supplemental Information 1 [file peerj-09-10672-s001.docx]

**Supplementary Information**

Widely assumed phenotypic associations in *Cannabis sativa* lack a shared genetic basis

Daniela Vergara^1*^, Cellene M. Feathers^1^, Ezra L. Huscher^1^, Ben Holmes^2^, Jacob Haas^1,3^, and Nolan C. Kane^1*^

**Results**

The MANOVA models with leaf shape as the response variable (PC1 and PC2, Table S6) suggest a within-leaf effect where these three traits ( leaf length, serration, and number of leaflets; Figure S1) are associated in the same leaf, but are not correlated between other leaves. However, when considering only PC1, leaf length (Figure S1A) shows a non-significant correlation. The deformation grids show that at shorter leaf lengths the middle leaflet is elongated and the outer leaflets are pointing downwards, while at longer leaf lengths the leaf tends to be squat with a smaller middle leaflet and the outer leaflets pointing out (Figure S1A). There is a marginally significant correlation between serration in the middle leaflet and leaf shape (PC1; Figure S1B). The deformation grids show that with fewer serrations the middle leaflet tends to be wider in the middle and the outer leaflets point downward. With numerous serrations the leaf is likely to be smaller with a narrower middle leaflet, and the outer leaflets tend to also be narrow and point outward. The number of leaflets and PC1 for shape are significantly correlated (Figure S1C) and the deformation grids illustrate that at fewer number of leaflets the leaves tend to be squat with the outer leaflets pointing outwards, while with numerous leaflets the leaves seem to be elongated with the outer leaflets pointing downwards.

While there was no relationship between cannabinoid level and leaf shape, there are correlations between the three measured cannabinoids (Figure S2). CBG is found in lower concentrations compared to THC or CBD which is expected given that CBGA is the precursor molecule to which THCA and CBDA synthases act on to convert to THCA and CBDA respectively. Therefore, if these synthases are acting efficiently, there should be little CBGA, which will be converted into CBG through heat. This is exactly what our results show (Figure S2). However, it is interesting that CBD is always present in lower levels compared to THC. Additionally, the negative correlation between THC and CBG (Figure S2C) signify that the high quantities of THC are associated to low quantities of CBG implying that THCA synthase is indeed converting CBGA into THCA. Our results also show that the three cannabinoids can coexist.

Another interesting trend observed with our data are the two clear chemotype groups (Figure S2). CBG is always found at lower quantities, but there are individuals that produce either high THC or high CBD, but not both. In other words, the individuals that produce high THC will produce low CBD and vice versa (Figure S2C), but CBG will always remain in low quantities (between 1-5% in both cases).
