# Supplementary figures and images for: Widely assumed phenotypic associations in Cannabis sativa lack a shared genetic basis
Source: PeerJ. 2021 Apr 20;9:e10672. doi: 10.7717/peerj.10672 (PMC8063869; doi:10.7717/peerj.10672)

**A**

PC1 (70.45% explained variation)

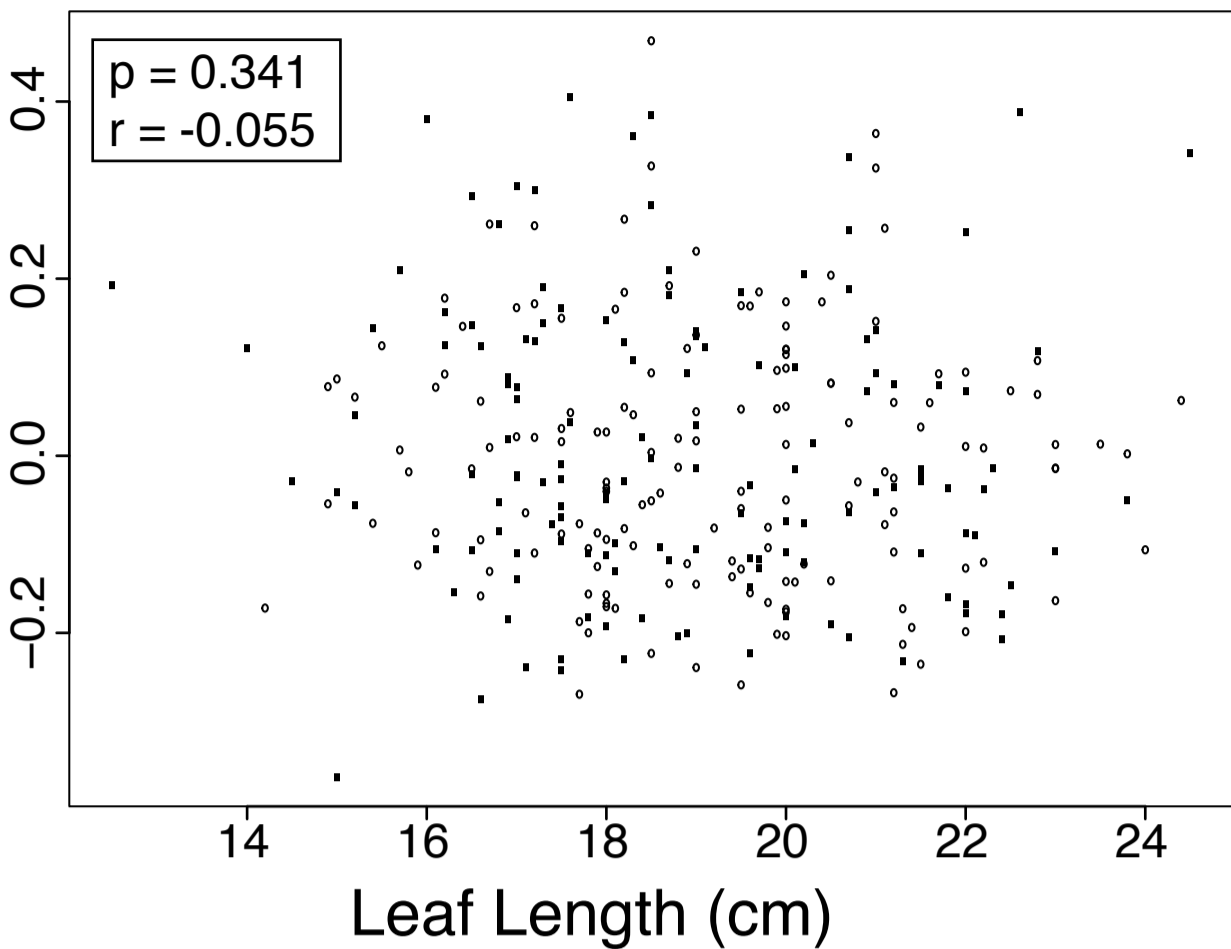

**B**

PC1 (70.45% explained variation)

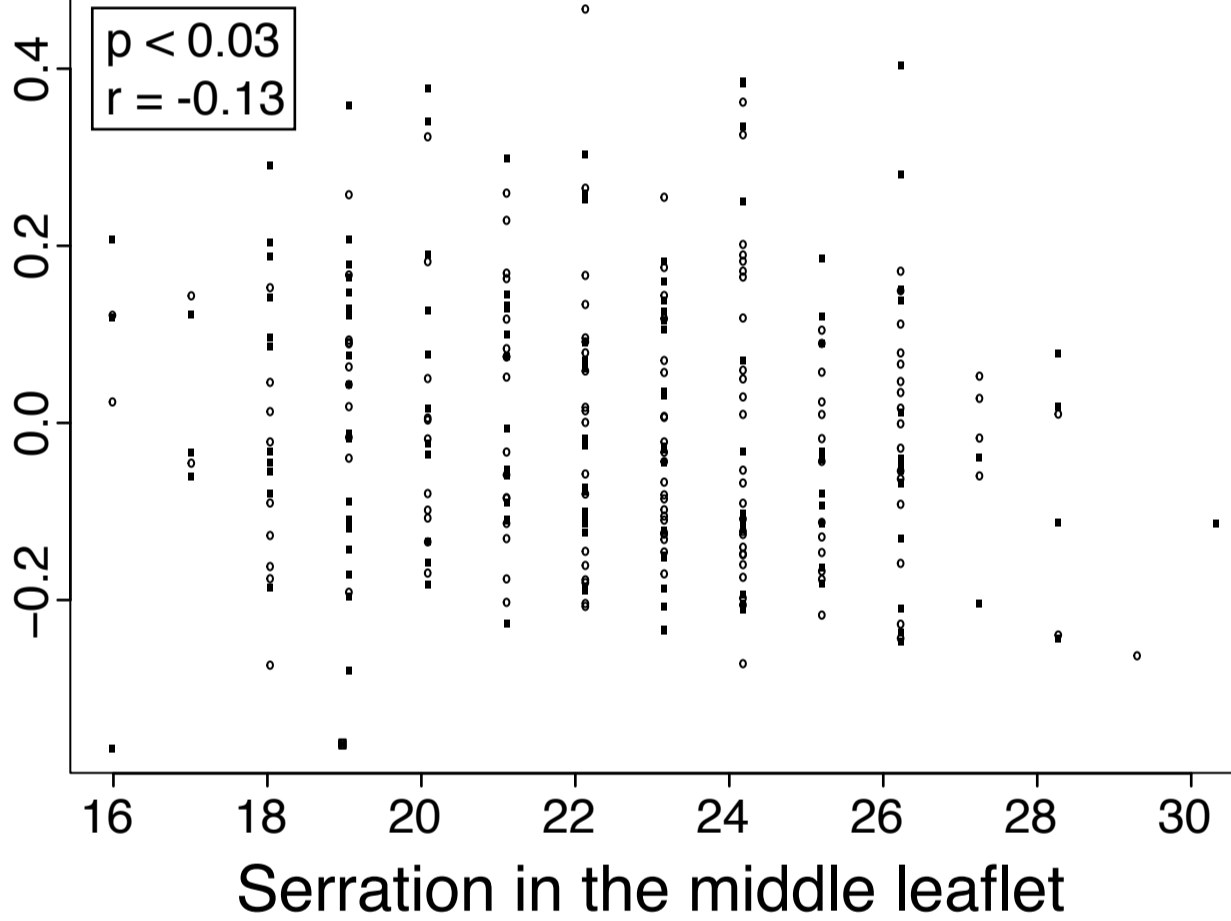

**C**

PC1 (70.45% explained variation)

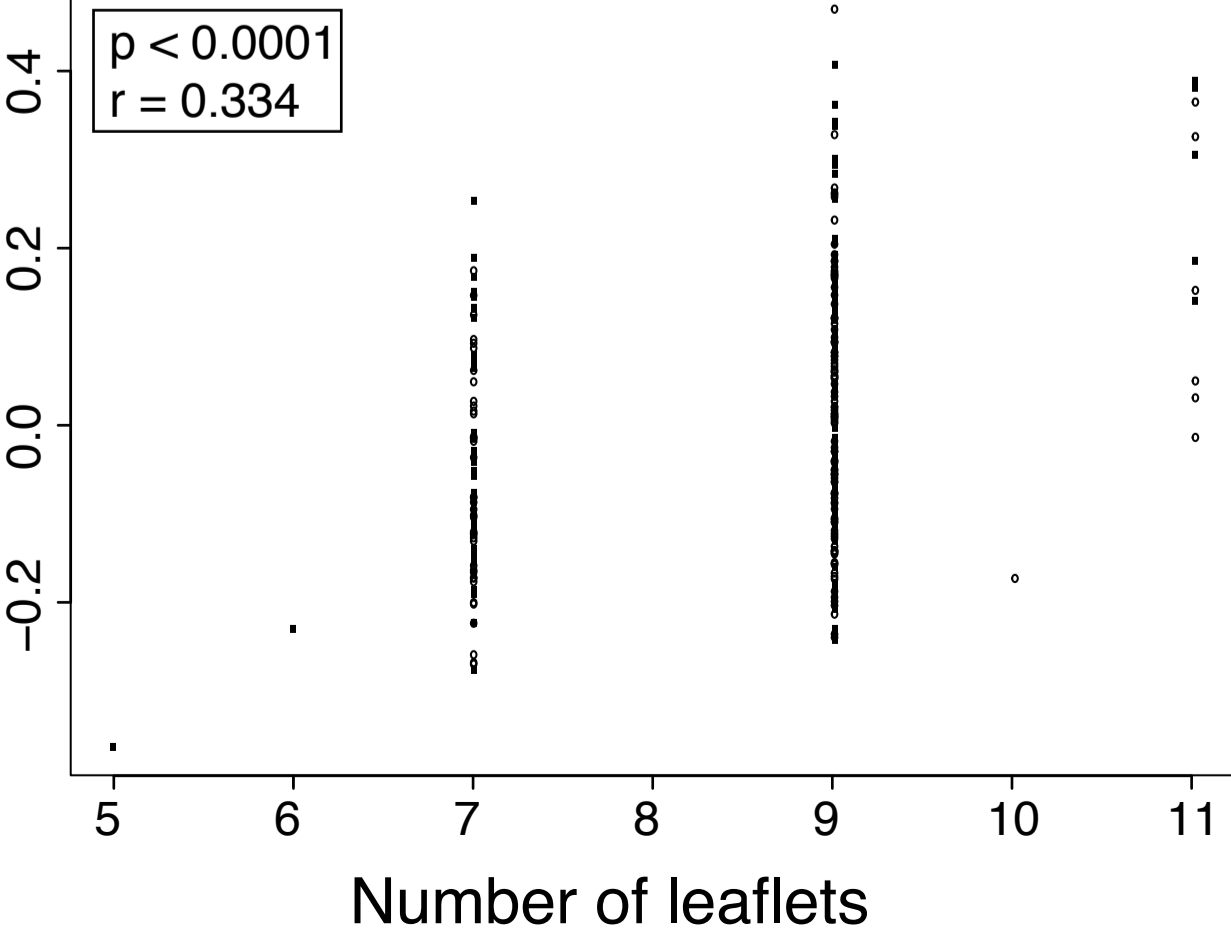

Supplement: Supplemental Information 2 — Leaf length is not correlated to PC1 (A), while serration in the middle leaflet (B) and number of leaflets (C) are marginally and significantly correlated, respectively. Each panel at the top has the deformation grids at the minimum (left) and maximum (right) values for length (A), serration in the middle leaflet (B), and number of leaflets (C). Males are shown in squares, females in open circles. [file peerj-09-10672-s002.pdf]

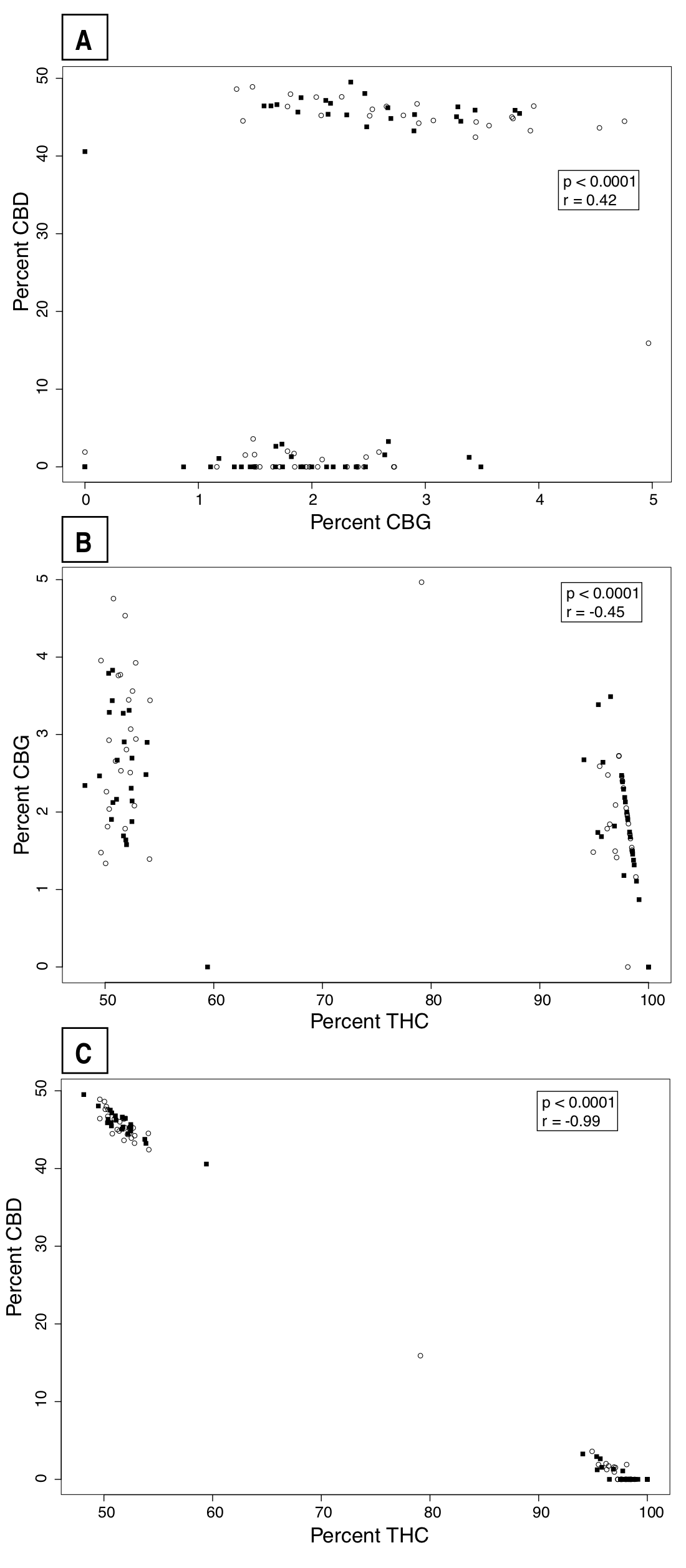

Supplement: Supplemental Information 3 — A and B show a positive correlation between CBG vs CBD, and THC vs CBG, respectively. C displays the negative linear relationship between THC and CBD. Notice the differences in the axis representing the disparity in production of the different cannabinoids. Males are shown in squares, females in open circles. [file peerj-09-10672-s003.png]
